# CD169/SIGLEC1 is expressed on circulating monocytes in COVID-19 and expression levels are associated with disease severity

**DOI:** 10.1007/s15010-021-01606-9

**Published:** 2021-04-06

**Authors:** Jan-Moritz Doehn, Christoph Tabeling, Robert Biesen, Jacopo Saccomanno, Elena Madlung, Eva Pappe, Frieder Gabriel, Florian Kurth, Christian Meisel, Victor M. Corman, Leif G. Hanitsch, Sascha Treskatsch, Kathrin Heim, Miriam S. Stegemann, Christoph Ruwwe-Glösenkamp, Holger C. Müller-Redetzky, Alexander Uhrig, Rajan Somasundaram, Claudia Spies, Horst von Bernuth, Jörg Hofmann, Christian Drosten, Norbert Suttorp, Martin Witzenrath, Leif E. Sander, Ralf-Harto Hübner

**Affiliations:** 1 Department of Infectious Diseases and Respiratory Medicine, Charité-Universitätsmedizin Berlin, corporate member of Freie Universität Berlin, Humboldt-Universität zu Berlin, and Berlin Institute of Health, 10117 Berlin, Germany; 2 Division of Pulmonary Inflammation, Charité-Universitätsmedizin Berlin, corporate member of Freie Universität Berlin, Humboldt-Universität zu Berlin, and Berlin Institute of Health, Berlin, Germany; 3 Berlin Institute of Health at Charité – Universitätsmedizin Berlin, Berlin, Germany; 4 Department of Rheumatology and Clinical Immunology, Charité-Universitätsmedizin Berlin, corporate member of Freie Universität Berlin, Humboldt-Universität zu Berlin, and Berlin Institute of Health, Berlin, Germany; 5 Department of Tropical Medicine, Bernhard Nocht Institute for Tropical Medicine, Hamburg, Germany; 6 Institute of Medical Immunology, Charité-Universitätsmedizin Berlin, corporate member of Freie Universität Berlin, Humboldt-Universität zu Berlin, and Berlin Institute of Health, Berlin, Germany; 7 Labor Berlin GmbH, Berlin, Germany; 8 Institute of Virology, Charité-Universitätsmedizin Berlin, corporate member of Freie Universität Berlin, Humboldt-Universität zu Berlin, and Berlin Institute of Health, Berlin, Germany; 9 German Centre for Infection Research (DZIF), Berlin, Germany; 10 Department of Anesthesiology and Intensive Care Medicine, Charité Campus Benjamin Franklin, Charité-Universitätsmedizin Berlin, corporate member of Freie Universität Berlin, Humboldt-Universität zu Berlin, and Berlin Institute of Health, Berlin, Germany; 11 Emergency Department, Charité-Universitätsmedizin Berlin, corporate member of Freie Universität Berlin, Humboldt-Universität zu Berlin, and Berlin Institute of Health, Berlin, Germany; 12 Department of Anesthesiology and Intensive Care Medicine, Charité Campus Mitte and Campus-Virchow-Klinikum, Charité-Universitätsmedizin Berlin, corporate member of Freie Universität Berlin, Humboldt-Universität zu Berlin, and Berlin Institute of Health, Berlin, Germany; 13 Department of Pediatric Pneumology, Immunology and Intensive Care Medicine, Charité-Universitätsmedizin Berlin, Berlin, Germany; 14 Associate member of the German Center for Lung Research (DZL), Marburg, Germany

**Keywords:** COVID-19, SARS-CoV-2, CD169, SIGLEC1, Type I interferons

## Abstract

Coronavirus disease 2019 (COVID-19) is caused by infection with severe acute respiratory syndrome coronavirus 2 (SARS-CoV-2). Type I interferons are important in the defense of viral infections. Recently, neutralizing IgG auto-antibodies against type I interferons were found in patients with severe COVID-19 infection. Here, we analyzed expression of CD169/SIGLEC1, a well described downstream molecule in interferon signaling, and found increased monocytic CD169/SIGLEC1 expression levels in patients with mild, acute COVID-19, compared to patients with severe disease. We recommend further clinical studies to evaluate the value of CD169/SIGLEC1 expression in patients with COVID-19 with or without auto-antibodies against type I interferons.

## Introduction

Coronavirus disease 2019 (COVID-19) is a pandemic viral infection caused by severe acute respiratory syndrome coronavirus 2 (SARS-CoV-2), initially observed in Wuhan, China at the end of 2019. Clinical manifestations of SARS-CoV-2 infection are highly variable ranging from asymptomatic infection to severe respiratory illness. Approximately, 5% of the patients develop severe COVID-19 with mortality rates of 50% due to acute respiratory distress syndrome (ARDS) and respiratory failure [1]. The pathomechanism of the deterioration is poorly understood, however, a dysregulation of inflammatory cell responses to SARS CoV-2 with a release of inflammatory cytokines described as “cytokine storm” is discussed [2, 3].

Type I interferons are known to balance the inflammatory host response to viruses and other diseases and have been suggested to play an important role in disease progression to severe COVID-19. Interferon signaling serves as an important defense system against virus infections since it supports virus clearance, activates tissue repair and regulates adaptive antiviral immune responses in the early infection. Recently, in about 10% of patients with severe COVID-19, neutralizing auto-antibodies against type I interferons were found but none in individuals with asymptomatic or mild disease [2]. Noteworthy, patients with auto-antibodies have almost undetectable plasma levels of type I interferons, suggesting neutralizing auto-antibodies and consecutive low interferon levels as important pathogenic factors for disease severity of COVID-19. In line, mutations in type I interferon related genes have been detected in 3.5% of patients with life-threatening COVID-19 pneumonia, and human fibroblasts holding type I interferon related gene mutations were tested vulnerable to SARS-CoV-2 [3].

Activation of the type I interferon pathway rapidly increases the expression of CD169 on the surface of monocytes and macrophages. CD169 is also termed sialic acid-binding immunoglobulin-like lectin 1 (SIGLEC1). CD169/SIGLEC1 expression levels correlate well with type I interferon levels, serving as an interferon signature [4]. Previous studies have demonstrated an important role of CD169/SIGLEC1 in different other viral infections, including Ebola virus and human immunodeficiency virus (HIV) [5, 6]. In agreement, CD169/SIGLEC1 expression has been proposed as a marker in the diagnosis of early COVID-19 [7].

As type I interferons play an important role in the early infection of COVID-19 and CD169/SIGLEC1 is known as a cell surface marker of interferon signaling, we longitudinally analyzed monocytic expression of CD169/SIGLEC1 in reverse transcription polymerase chain reaction (RT-PCR)-confirmed patients with mild and severe COVID-19 in comparison to RT-PCR-confirmed control patients with other diseases. Our results support several transcriptome-based studies indicating a waning interferon signature in COVID-19 patients over time [8]. Strikingly, this interferon signature was absent in patients with severe COVID-19 compared to mild disease. Hence, CD169/SIGLEC1 is linked to the early phase of mild COVID-19 infection and lower expression levels may account for the severity of the disease. These observations highlight interferon signaling as an important player in the development of life threatening ARDS in COVID-19.

## Methods

At Charité-Universitätsmedizin Berlin, Germany, from March 18 to December 07, 2020, 603 ETDA blood samples were analyzed from 129 patients with suspected SARS-CoV-2 infection who were retrospectively enrolled for this study. Inclusion criteria for this study were hospitalization, either on a normal ward or in an intensive care unit, COVID-19 suspected symptoms as fever, acute respiratory symptoms, diarrhea and/or loss of smell or taste dysfunction and the assessment of CD169/SIGLEC1 on monocytes. Exclusion criterion was an indefinable onset of symptoms. All patients were tested for SARS-CoV-2 infection by nasopharyngeal swabs or bronchoalveolar lavage using real-time RT-PCR as recently described [9]. Based on extensive clinical evaluation, computed tomography of the lungs and broad laboratory testing, a definite diagnosis could be achieved for each patient. Clinical characteristics and laboratory findings were obtained from electronic medical records. The study was approved by local ethics (EA2/252/20 and EA2/066/20). COVID-19 patients were stratified according to disease severity using the WHO ordinal scale for clinical improvement (www.who.int). For this study, we defined the following groups:

1. 51 hospitalized patients with mild COVID-19 tested positive for SARS-CoV-2 RNA. Mild disease was characterized by hospitalisation and no oxygen therapy (WHO group 3) or oxygen therapy by mask or nasal prongs (WHO group 4).
2. 51 hospitalized patients with severe COVID-19 tested positive for SARS-CoV-2 RNA. Severe disease was characterized by non-invasive ventilation or high-flow oxygen (WHO group 5), intubation and mechanical ventilation (WHO group 6) or ventilation with an additional organ support as renal replacement therapy or extracorporeal membrane oxygenation (WHO group 7).
3. 27 patients with exclusion of COVID-19 based on negative SARS-CoV-2 RT-PCR testing, computed tomography or X-ray of the chest, laboratory test results and clinical course. Specifically, these SARS-CoV-2-negative patients were diagnosed with community acquired pneumonia (*n* = 12), acute exacerbation of chronic obstructive pulmonary disease (*n* = 4), acute tracheobronchitis (*n* = 4), pyelonephritis (*n* = 2), sarcoidosis (*n* = 1), myocardial infarction (*n* = 1), pulmonary artery embolism (*n* = 1), acute exacerbation of asthma (*n* = 1) and acute kidney failure (*n* = 1).

In all patients, CD169/SIGLEC1 expression on monocytes was analyzed based on a method described previously [10]. In brief, CD169/SIGLEC1 expression on monocytes was analyzed in EDTA whole blood. Whenever possible, analyses were performed on the same day within 4 h after blood samples were taken. Otherwise, EDTA blood was stored at 2–8 °C and immediately processed the next morning. Of note, CD169/SIGELC-1 expression on monocytes in stored EDTA blood (at 2–8 °C for not longer than 24 h) differed less than 10% compared to baseline for freshly obtained blood. Analysis was performed using an accredited flow cytometry protocol at the clinical diagnostics laboratory (Labor Berlin GmbH). Samples were incubated with a mouse anti-human antibody mixture consisting of antibodies against CD169/SIGLEC1 (clone 7–239), CD14 and CD45 (all antibodies were obtained from Beckman Coulter). In hospitalized COVID-19 patients, sequential blood sampling was obtained in 79 patients. In 23 patients only single sampling was performed as they were early dismissed from the hospital.

Quantitative viral loads in nasopharyngeal swab samples were available in 35 patients diagnosed with COVID-19. SARS-CoV-2 RT-PCR results were obtained using respiratory samples taken for routine testing and using two test system. First, we used an assay targeting the SARS-CoV-2 E-gene and second, we used the cobas® SARS-CoV-2 test on the cobas® 6800/ 8800 system. The assessment of SARS-CoV-2 RNA concentration was done by applying external calibration curves and quantified in-vitro transcribed RNA to assess the complete SARS-CoV-2 RNA. SARS-CoV-2-specific immunoglobulin G (IgG) levels were assessed in 31 confirmed COVID-19 cases as described previously [9]. Measurement was done by anti-SARS-CoV-2 S1 IgG ELISAs (Euroimmun AG, Lübeck, Germany) following manufacturer's instructions. Statistical analyses were performed using GraphPad Prism 8. For comparison of monocytic CD169/SIGLEC1 expression levels between groups, Kruskal Wallis test was performed followed by Dunn’s multiple comparison test. In cases of positive SARS-CoV-2 RT-PCR results, correlation analyses between viral load and CD169/SIGLEC1 expression levels (measured within the same time period ± 2 days) were performed via Pearson correlation, and linear regression was calculated.

## Results

CD169/SIGLEC1 expression levels on monocytes were evaluated in 51 hospitalized patients with mild COVID-19 (33/18 male/female, 57.1 ± 2.2 years of age), 51 hospitalized patients with severe COVID-19 (37/14 male/female, 62.5 ± 2.0 years of age) and 27 patients (14/13 male/female, 63.5 ± 3.93 years of age) with exclusion of COVID-19.

**Fig. 1:**
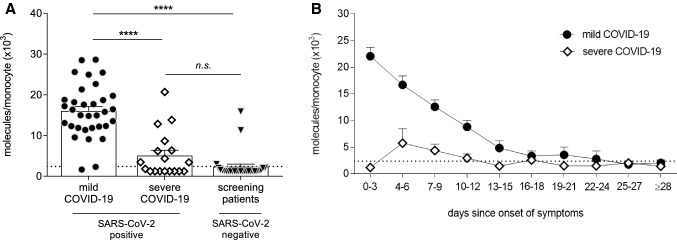
Expression analysis of sialic acid-binding immunoglobulin-like lectin 1 (CD169/SIGLEC1) on circulating monocytes in patients with mild and severe coronavirus disease 2019 (COVID-19). In the early phase of infection with severe acute respiratory syndrome coronavirus 2 (SARS-CoV-2), 0–9 days after onset of symptoms, CD169/SIGLEC1 expression was strongly upregulated in patients with mild COVID-19 (*n* = 33) compared to patients with severe COVID-19 (*n* = 18), and compared to COVID-19 screening patients tested negative for SARS-CoV-2 (*n* = 27) (**a**). Longitudinal analyses revealed that monocytic CD169/SIGLEC1 expression in patients with mild COVID-19 (*n* = 51) gradually decreased over time and eventually reached the normal range as indicated by the dotted line (< 2400 molecules/monocyte) (**b**). In patients with severe COVID-19 (*n* = 51), CD169/SIGLEC1 expression undulated around the upper limit of the normal range (**b**). CD169/SIGLEC1 expression was assessed via flow cytometry with a detection limit of 1200 molecules/monocyte. Mean values are shown if patients were repetitively tested within the same time period (**a**, **b**). Data are shown as mean + standard error of mean (SEM) (**a**, **b**). For A, one symbol represents one patient. For comparison between groups, Kruskal Wallis test was performed followed by Dunn’s multiple comparison test, ****p ≤ 0.0001, n.s. = not significant

Since in COVID-19, the median duration from onset of symptoms to ARDS is 9 days [1], we first compared CD169/SIGLEC1 expression levels in this early phase of infection. Within this range, 0–9 days after onset of symptoms, patients with mild COVID-19 showed an increased CD169/SIGLEC1 expression compared to patients with severe COVID-19, and also compared to SARS-CoV-2 negative patients (*p* < 0.0001 for both comparisons, Fig. 1a). Interestingly, CD169/SIGLEC1 levels in patients with severe COVID-19 were similar to SARS-CoV-2 negative patients (Fig. 1a).

In line with the literature that CD169/SIGLEC1 expression is not specific for COVID-19, three out of 27 COVID-19 screening patients tested negative for SARS-CoV-2 showed CD169/SIGLEC1 expression levels above the normal range. These patients suffered from fever associated with rheumatoid arthritis, bacterial respiratory infection in the context of preexisting lung fibrosis, and tracheobronchitis.

Among patients with mild COVID-19, there was a time-dependent expression of CD169/SIGLEC1 with the highest values within the first 3 days after onset of symptoms (Fig. 1b). Then, expression levels decreased to normal ranges within 3–4 weeks after onset of symptoms. In contrast, patients with severe COVID-19 showed almost normal expression levels of CD169/SIGLEC1 (Fig. 1b).

**Fig. 2:**
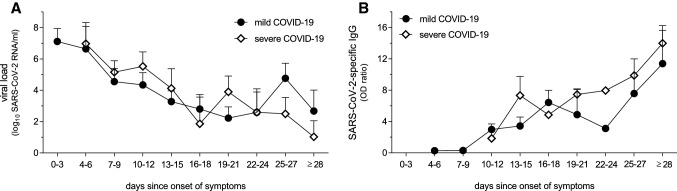
Longitudinal SARS-CoV-2-specific viral load (**a**) and anti-SARS-CoV-2 IgG concentrations (**b**) in COVID-19 patients. Both the gradual decrease in vial load (**a**) and the seroconversion (**b**) were similar in patients with mild and severe COVID-19. Mean values are shown if patients were repetitively tested within the same time period (**a**, **b**). Data are shown as mean + SEM, n = 10–25 (**a**), *n* = 10–21 (**b**)

To exclude that the here observed low CD169/SIGLEC1 expression levels in patients with severe COVID-19 may be the consequence of an altered viral load, SARS-CoV-2 viral load was quantified at different time points as shown before [9]. Similar peak concentrations were observed within the first week for all patients with mild and severe COVID-19, and viral load then gradually decreased below detection limit within 4 weeks (Fig. 2a). In accordance to viral load, seroconversions were similar in patients with mild and severe COVID-19 (Fig. 2b). In both COVID-19 groups, SARS-CoV-2-specific IgG was detectable 10–12 days after onset of symptoms (Fig. 2b).

**Fig. 3:**
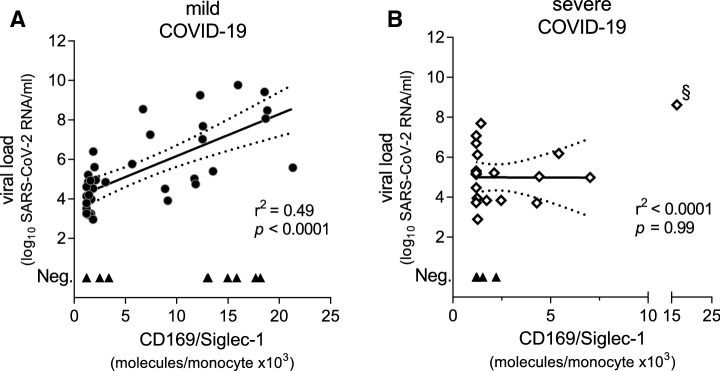
SARS-CoV-2 specific viral load correlated with CD169/SIGLEC1 expression levels in patients with mild COVID-19 disease (**a**) in contrast to severe COVID-19 disease (**b**). Mean values are shown if patients were repetitively tested within the same time period. All positive viral loads were included in further analyses, except one significant outlier (as indicated by §) (**b**). Pearson correlation was performed and linear regression was calculated; the dotted lines represent the 95% confidence band, *n* = 25 (**a**), *n* = 10 (**b**)

To analyze for possible associations between CD169/SIGLEC1 expression levels and disease severity of COVID-19, we performed Pearson correlations. In patients with mild disease, CD169/SIGLEC1 expression levels strongly correlated with the viral load (*r*^2^ = 0.49, *p* < 0.0001, Fig. 3a). This correlation, however, was absent in patients with severe disease (*r*^2^ =  < 0.0001, *p* = 0.99, Fig. 3b).

## Discussion

In this retrospective observational study, CD169/SIGLEC1 expression was upregulated on circulating monocytes of COVID-19 patients with mild disease. Exclusively, these patients revealed an early peak within 3 days after onset of symptoms and demonstrated a gradual decrease down to normal levels within the next 3–4 weeks. In contrast, CD169/SIGLEC1 expression in most COVID-19 patients with severe disease ranged in almost normal levels comparable with screening patients following exclusion of COVID-19, although control patients were not matched in regard to sex.

However, it is not clear how CD169/SIGLEC1 expression on monocytes is regulated by SARS-CoV-2. The immunoglobulin-like domains of CD169/SIGLEG1 are known to play an important role in the antiviral and antibacterial host response, as well as in the pathogenesis of autoimmune and other diseases. In HIV infection for instance, monocytic CD169/SIGLEC1 expression is highly upregulated in the early phase of infection between day 5 and day 15 [4].

Although not fully understood, induction of CD169/SIGLEC1 expression levels were found to be regulated by cytokines such as type I interferons [4, 6]. Type I interferons are crucial for the immune response in antiviral immunity. It has been reported that an unbalanced immune response, characterized by a weak release of type I interferons and an exaggerated release of other proinflammatory cytokines, potentially contributes to a SARS-CoV-2-associated ARDS. In agreement with this observation, we exclusively found higher expression levels of CD169/SIGLEC1 in mild disease stages but not in severe forms, suggesting that CD169/SIGLEC1 or other interferon-associated downstream mediators are protective in disease progression to ARDS.

The here observed elevated CD169/SIGLEC1 expression levels in COVID-19 patients are in line with a previous report by Bedin and colleagues [7], who first described elevated CD169/SIGLEC1 expression in a small cohort of patients with COVID-19, but no differences in CD169/SIGLEC1 expression levels were observed between mild and severe cases. Our data, however, for the first time, show a clear disease severity-dependent expression of CD169/SIGLEC1.

Noteworthy, the very recent discovery of auto-antibodies against type I interferons in about 10% of patients with severe COVID-19 and the presence of mutations in type I interferon related genes in 3.5% of patients with severe COVID-19 shed an exciting light on a possible interferon-related pathomechanism of disease severity [2, 3]. While detection of auto-antibodies and/or mutations might be difficult to establish as routine diagnostics, surrogate markers of interferon type I activity as CD169/SIGLEC1 may be favorable.

Our results of viral load and seroconversion indicate representative COVID-19 patient samples, although it is important to note that data were not available for all patients as a consequence of the retrospective study design. In addition, there are more important limitations to consider. Although CD169/SIGLEC1 is suggested to have an additional diagnostic value in COVID-19, it remains unclear whether expression levels represent a bystander or a direct contributor to the pathogenesis of COVID-19. Our longitudinal data suggest that the early phase after onset of symptoms is the best timepoint to assess CD169/SIGLEC1. However, numbers of patients presenting to the hospital in this very early time period, within the first 3 days since onset of symptoms, is rather low. Furthermore, the number of patients with mild disease who developed a deterioration to a more severe disease stages was insufficient to answer the question whether CD169/SIGLEC1 may be used as a biomarker to predict the progression to ARDS. Nevertheless, it will be interesting to see whether treatment or vaccination trials will have an impact on CD169/SIGLEC1 expression levels after SARS-CoV-2 infection or exposition respectively. Also, it is interesting to analyze the correlation between CD169/SIGLEC1 expression and auto-antibodies against IFN-α/ω in patients with mild or severe disease. Certainly, other molecules of the interferon pathways as MHC class I, CD38, BST-2 or CD123 should be analyzed in patients with COVID-19.

Taking these limitations into account, our study provides important new insights in the dynamics of CD169/SIGLEC1 expression in SARS-CoV-2 infection. Furthermore, our findings suggest that measurement of CD169/SIGLEC1 expression in SARS-CoV-2 infection might be useful for assessment of COVID-19 disease severity in the future. However, for more clarification, we suggest a prospective study with larger patient cohorts and a more balanced control group to confirm our results.

## Abbreviations

ARDS: Acute respiratory distress syndrome
COVID-19: Coronavirus disease 2019
IgG: Immunoglobulin G
RT-PCR: Reverse transcription polymerase chain reaction
SARS-CoV-2: Severe acute respiratory syndrome coronavirus 2
SIGLEC1: Sialic acid-binding immunoglobulin-like lectin 1
